# Predicting the Lymphovascular Invasion in Carcinoma Bladder at Transurethral Resection

**DOI:** 10.7759/cureus.62595

**Published:** 2024-06-18

**Authors:** Anil Kumar Nallabothula, Harsha Vardhana Varma Mudunuri, Anirudh Suseel Nalumaru, Viswanth Kodamanchile, Sai Bhashya Vamsi Krishna Varanasi, Naveen Kumar Yadlapalli, Dinesh Earla

**Affiliations:** 1 Department of Urology, Sri Venkateswara Institute of Medical Sciences, Tirupati, IND

**Keywords:** high-grade urothelial carcinoma, hematuria, lymphovascular invasion, histopathology examination, turbt, bladder cancer

## Abstract

Introduction: Bladder cancer is among the most common malignant neoplasms in the world. Transurethral resection of bladder tumor (TURBT) is considered the standard procedure for diagnosis, staging, and risk classification of bladder tumors. Lymphovascular invasion (LVI) is considered a poor prognostic factor. Its assessment of TURBT is very important for risk stratification and decision-making for further treatment. The purpose of our clinical study is to attempt to predict/assess the correlation between LVI and various preoperative (age, gender, history of smoking, hematuria, urine cytology, and hydronephrosis/hydroureteronephrosis), intraoperative (tumor number, size, and appearance - sessile/ pedunculated) and histopathological (tumor histology, grading, and muscle invasion) factors.

Methodology: In this prospective study, 75 patients with bladder tumors underwent TURBT (standard monopolar TURBT with 1.5% glycine as irrigation solution) in the Department of Urology at Sri Venkateswara Institute of Medical Sciences (SVIMS), Tirupati between October 2021 and March 2023. Histopathological examination (HPE) reports were looked for the presence or absence of LVI. Accordingly, patients were divided into two groups, i.e., those with LVI and those without LVI. Various preoperative and intraoperative variables were analyzed for each subject in both groups. Statistically significant variables occurring in those patients with LVI compared to those without LVI were considered predictors of LVI in bladder tumors.

Results: Sixteen patients out of 75 (21.33%) had LVI on their histopathology examination. The mean age was 68.19 years in the group with LVI and 64.14 years in the group without LVI. A total of 60 men (80%) and 15 women (20%) were included in our study. Thirteen men (21.7%) and three women (20%) were found to have LVI. We observed a significant association between the appearance of the tumor and LVI. Fifty-four subjects in our study had sessile tumors. Fifteen out of them (27.8%) had LVI, while only one out of 21 patients (4.8%) with pedunculated tumors had LVI (p-value=0.028). 30% of subjects who had high-grade tumors on HPE also had LVI. On the contrary, only one of 25 patients (4%) with low-grade tumors had LVI (p-value=0.010). Our study also showed a significant association between muscle invasion and LVI. Thirty-four (45.3%) and 41 (54.7%) patients had muscle-invasive and non-muscle-invasive tumors, respectively. While 12 (35.3%) patients with muscle-invasive tumors had LVI, only four (9.8%) patients with non-muscle-invasive tumors showed LVI (p-value=0.007).

Conclusion: We observed that LVI of bladder tumors at first TURBT is significantly associated with tumor grade, tumor appearance, and depth of invasion of the tumor. Though statistically not significant, we further observed that LVI was more commonly found in smokers, patients with hematuria, and larger tumor sizes. We conclude that these factors can be used as reliable predictors of LVI of bladder tumors at their first TURBT.

## Introduction

Bladder cancer is among the most common malignant neoplasms in the world. Its contribution is 3.4% to the total cancer burden worldwide and is ranked 10th among all cancers in the world. In India, the incidence rate is 2.4 and 0.7 per 100,000 males and females, respectively, and the mortality rate is 1.3 and 0.3 per 100,000 males and females, respectively [1].

TURBT is considered the standard procedure for the diagnosis, staging, and risk classification of bladder tumors. Tumors are generally staged according to the AJCC/TNM staging system [2]. On average, 70% of the cases newly diagnosed as bladder carcinoma are non-muscle-invasive bladder cancer (NMIBC - limited to mucosa or lamina propria). The World Health Organization (WHO)/International Society of Urological Pathology (ISUP) presently recommends classifying malignant tumors as low-grade or high-grade regardless of invasion status. Lymphovascular invasion (LVI) is the invasion of cancer cells within any lymphatic, arterial, or venous lumen. LVI is considered a poor prognostic factor, and it is classified under the high-risk category of NMIBC. The evidence strength about the prognostic value of LVI in TURBT is grade C and its recommendation is moderate [3]. LVI has been established as an independent predictor of recurrence and decreased cause-specific and overall survival in patients who undergo cystectomy for invasive bladder cancer and are node-negative. [4].

The assessment of LVI on TURBT and report­ing is very important for risk stratification and decision-making for further treatment. Being able to predict LVI before a first TURBT will help the urologist in consciously performing intensified TURBT. Additionally, it is considered very useful for sched­uling more aggressive follow-up protocols and further treatments in routine clinical practice. This prospective study was performed at Sri Venkateswara Institute of Medical Sciences (SVIMS), Tirupati with an aim to identify factors that could predict LVI before/at TURBT. Various pre-operative factors viz., age, gender, history of smoking, hematuria, urine cytology, and hydronephrosis on imaging; and intraoperative factors viz., tumor size, tumor number, and appearance of the tumor - sessile/pedunculated, histology, and muscle invasion were looked for their association with LVI.

## Materials and methods

This prospective study was performed after receiving approval from the institutional ethics committee (IEC No. 1218 dated 29/09/2021) and scientific committee. A total of 75 patients with bladder tumors who underwent TURBT for the first time between October 2021 and March 2023 were included in this study after obtaining written informed consent. Those unwilling and medically unfit for the procedure, patients with recurrent bladder tumors, and those undergoing re-TURBT were excluded from the study.

All the patients with suspected bladder tumors from a thorough history and physical examination were subjected to investigations to establish the diagnosis and aid in surgery. These included surgical profile (complete blood picture, renal function test, serum electrolytes, blood grouping, Rh typing, urine routine, microscopy, urine culture, sensitivity, and urine cytology) and special investigations to establish the location and extent of the tumor, which include ultrasonography (USG) of the abdomen and pelvis or computerized tomography (CT) of the abdomen and pelvis and cystoscopy when required. The Paris System (TPS) was used for reporting urine cytology.

Once the patients were diagnosed to have a bladder tumor, he/she was subjected to the surgical procedure (TURBT). The procedure involved putting the patient in a lithotomy position under regional or general anesthesia. Resection was performed using a 30-degree endoscopic lens (KARL STORZ SE & Co., Tuttlingen, Germany) passed through a 26Fr resectoscope sheath (KARL STORZ SE & Co., Tuttlingen, Germany) under continuous bladder irrigation with 1.5% glycine. Monopolar diathermy connected to a cutting loop was used to facilitate resection of the tumor and coagulation of the bleeding points. Resection was performed piecemeal. After all the visible tumors had been resected, an additional pass of the cutting loop was done to resect the tumor base and this tissue was sent for pathological examination separately. After ensuring adequate hemostasis, a 20Fr 3-way latex Foley’s catheter was inserted and irrigation started with normal saline.

Histopathological examination (HPE) was performed by an experienced onco-pathologist. Routinely, tissues were fixed with neutral formalin 10%, embedded in paraffin, and then manually sectioned with a microtome to obtain 4-5 µm thick paraffin sections. Dewaxed sections were then stained with Hematoxylin & Eosin (H&E) for HPE. Sections were looked for the presence of the tumor, its histology, grade and differentiation, and depth of invasion. Additionally, the presence/absence of LVI and perineural invasion were also looked for. Accordingly, patients were divided into two groups, i.e., those with LVI and those without LVI. All the preoperative and intraoperative variables were analyzed for each subject in both groups. Statistically significant variables occurring in those patients with LVI compared to those without LVI were considered predictors of LVI in bladder tumors. 

Statistical analysis was performed using Statistical Package for Social Sciences (SPSS) for Windows software (version 22.0; SPSS Inc., Chicago). Descriptive statistics such as mean and standard deviation (SD) for continuous variables and frequencies and percentages for categorical variables were determined. Continuous parametric variables were compared using t-tests. Non-parametric variables were compared using the Mann-Whitney U test. Categorical variables were compared using the Chi-square test or Fisher’s exact tests. Associations between LVI and preopera­tive variables were assessed by univariate analysis. Bar charts and pie diagrams were used for visual representation of the analyzed data. P-value <0.05 was considered statistically significant.

## Results

**Figure 1:**
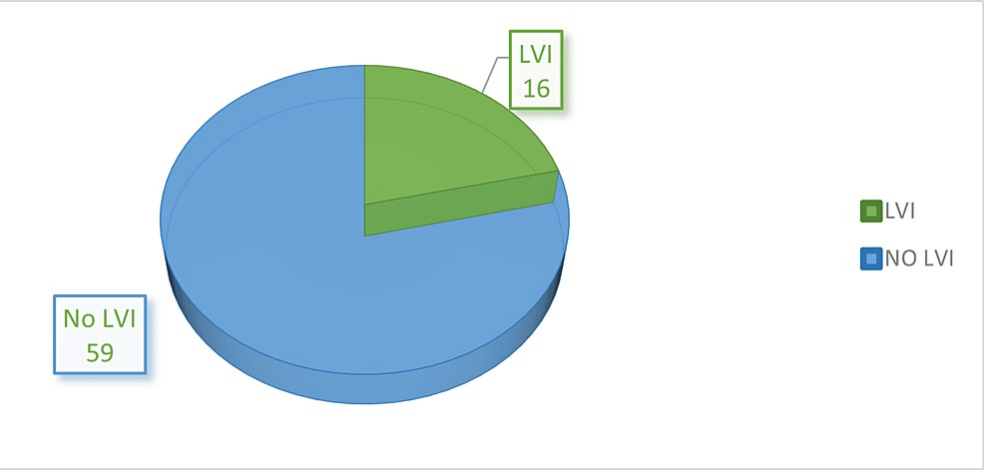
Distribution of patients LVI, lymphovascular invasion

Out of the total 75 patients, 16 (21.33%) had LVI on histopathology examination while 59 (78.67%) had no LVI (Figure 1).

The mean age was 68.19 years in the group with LVI and 64.14 years in the group without LVI with SDs of 11.61 and 11.81, respectively. The p-value was 0.113, which was statistically not significant. We had a total of 60 men and 15 women who were included in the study. Thirteen men and three women were found to have LVI. History of smoking and hematuria were not found to be significantly associated with LVI in our study (p-value of 0.360 and 0.475, respectively) (Table 1).

**Table 1:** Baseline characteristics of the patients LVI, lymphovascular invasion

		With LVI (n=16)	No LVI (n=59)	Total (n=75)	P-value
Age (years)	Mean (SD)	68.19 (11.61)	64.14 (11.81)	65.00 (11.81)	0.113
Gender	Male	13 (21.7%)	47 (78.3%)	60 (80%)	0.888
	Female	3 (20%)	12 (80%)	15 (20%)	
Smoking	Yes	12 (24.5%)	37 (75.5%)	49 (65.3%)	0.360
	No	4 (15.4%)	22 (84.6%)	26 (34.7%)	
Hematuria	Yes	14 (23%)	47 (77%)	61 (81.3%)	0.475
	No	2 (14.3%)	12 (85.7%)	14 (18.7%)	

In our study, 26 patients (34.7%) of the total study population had high-grade urothelial malignancy in their urine cytology. Only eight (30.7%) of them had LVI. Likewise, most patients from the other categories of urine cytology had no LVI. Fifty-seven (76%) of the total study population had no HDN/HDUN on imaging studies. While 10 patients (17.5 %) with no HDN/HDUN had LVI, only six patients (33.3%) with HDN/HDUN had LVI. This association was statistically not significant with a P-value of 0.154 (Table 2).

**Table 2:** Association of urine cytology and hydronephrosis with LVI LVI, lymphovascular invasion

		With LVI (n=16)	No LVI (n=59)	Total (n=75)	P-value
Urine cytology	Atypical cells	2 (12.5%)	14 (87.5%)	16 (21.3%)	0.465
	High grade	8 (30.7%)	18 (69.3%)	26 (34.7%)	
	Low grade	-	5 (100%)	5 (6.7%)	
	Negative	5 (22.7%)	17 (77.3%)	22 (29.3%)	
	Suspicious of high grade	1 (16.7%)	5 (83.3%)	6 (8%)	
Hydronephrosis	Present	6 (33.3%)	12 (66.7%)	18 (24%)	0.154
	Absent	10 (17.5%)	47 (82.5%)	57 (76%)	

**Figure 2:**
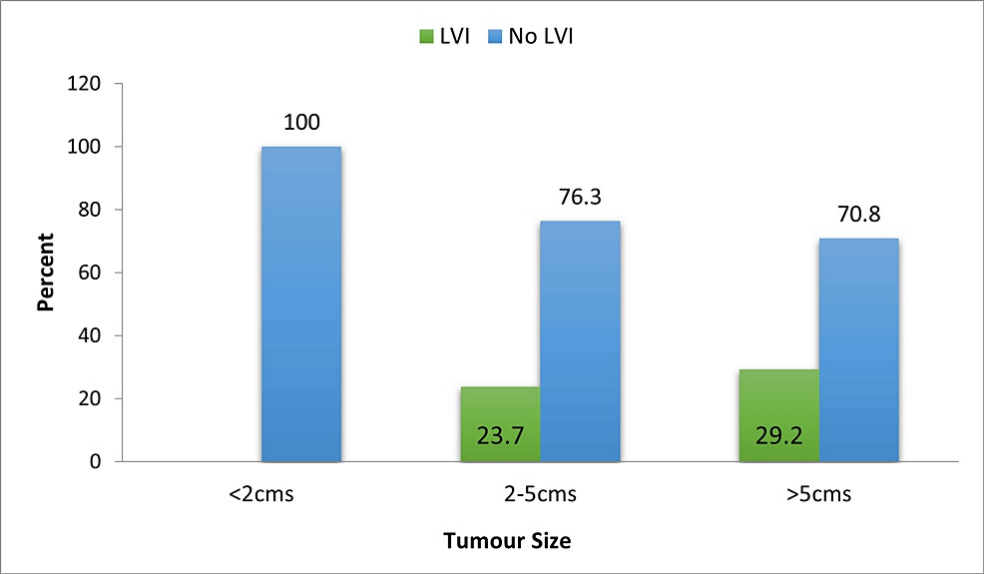
Association between tumor size and LVI LVI, lymphovascular invasion

**Figure 3:**
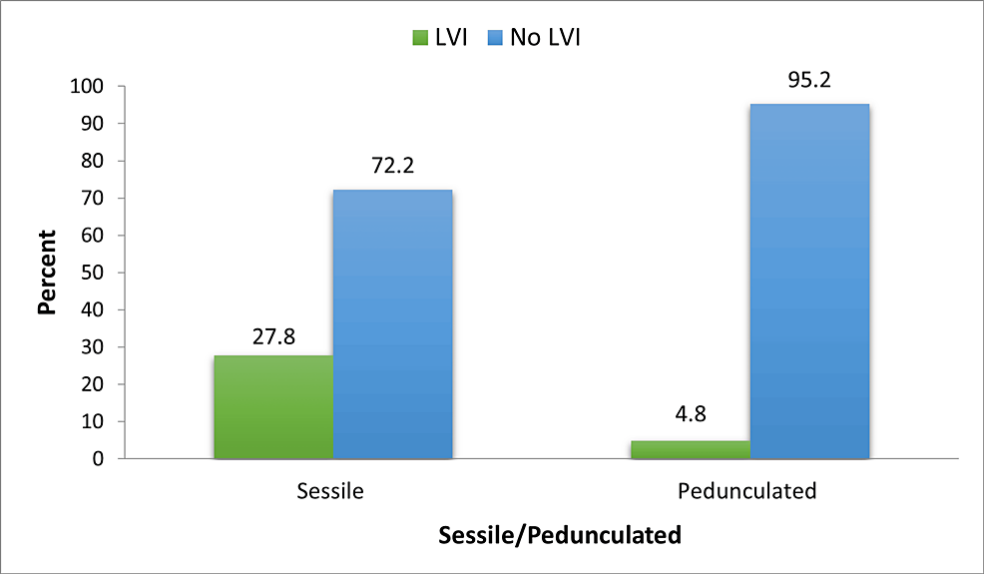
Association between tumor appearance and LVI LVI, lymphovascular invasion

Fifty patients (66.7%) had solitary tumors and 25 (33.3%) patients had multiple tumors. With a p-value of 0.842, the tumor number had no statistically significant association with the incidence of LVI, as seen in Table 3. Just over half of the study subjects (50.7%) had tumor sizes ranging from 2-5 cm. No tumor of size less than 2 cm had LVI on HPE. We observed that the incidence of LVI increased as the tumor size increased, but this association was not statistically significant (p-value=0.104) (Figure 2 and Table 3). Most patients with either sessile or pedunculated tumors had no LVI on their HPE. Fifteen (27.8%) out of 54 patients with sessile tumors had LVI. In comparison only one (4.8%) patient out of 21 with pedunculated tumors had LVI. This association was noted to be statistically significant with a p-value of 0.028 (Figure 3 and Table 3).

**Table 3:** Association between tumor characteristics and LVI LVI, lymphovascular invasion

		With LVI (n=16)	No LVI (n=59)	Total (n=75)	P=value
Tumor number	Single	11 (22%)	39 (78%)	50 (66.7%)	0.842
	Multiple	5 (20%)	20 (80%)	25 (33.3%)	
Tumor size	<2 cm	-	13 (100%)	13 (17.3%)	0.104
	2-5 cm	9 (23.7%)	29 (76.3%)	38 (50.7%)	
	>5 cm	7 (29.2%)	17 (70.8%)	24 (32%)	
Tumor appearance	Sessile	15 (27.8%)	39 (72.2%)	54 (72%)	0.028
	Pedunculated	1 (4.8%)	20 (95.2%)	21 (28%)	

We had 66 patients (88%) who had transitional cell carcinoma (TCC) on their HPE. Fifteen of these patients had LVI while 51 had no LVI. This association was statistically not significant (p-value=0.948). Five other patients had TCC with squamous differentiation. Only one patient had LVI and the remaining four had no LVI (statistically not significant). The other histologies we found with one patient in each category were adenocarcinoma, papillary urothelial neoplasm of low malignant potential (PUNLMP), TCC with glandular differentiation, and well-differentiated squamous cell carcinoma (SCC). None of them had LVI (Table 4).

**Table 4:** Association between tumor histology and LVI LVI, lymphovascular invasion; TCC, transitional cell carcinoma; PUNLMP, papillary urothelial neoplasm of low malignant potential; SCC, squamous cell carcinoma

		With LVI	No LVI	Total	P-value
Histology	Adenocarcinoma	-	1 (100%)	1 (1.3%)	0.948
	PUNLMP	-	1 (100%)	1 (1.3%)	
	TCC	15 (22.7%)	51 (77.3%)	66 (88%)	
	TCC with glandular differentiation	-	1 (100%)	1 (1.3%)	
	TCC with squamous differentiation	1 (20%)	4 (80%)	5 (6.7%)	
	SCC	-	1 (100%)	1 (1.3%)	

**Figure 4:**
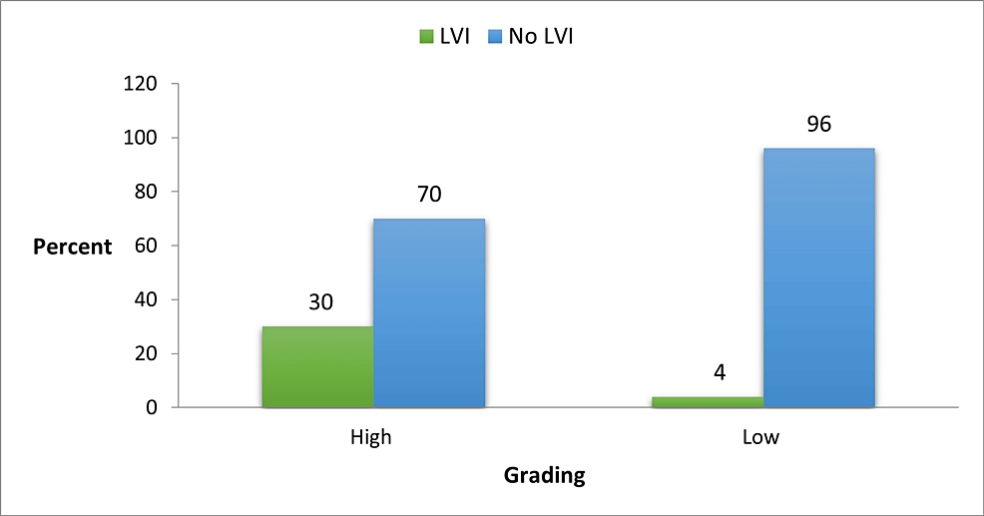
Association between tumor grade and LVI LVI, lymphovascular invasion

**Figure 5:**
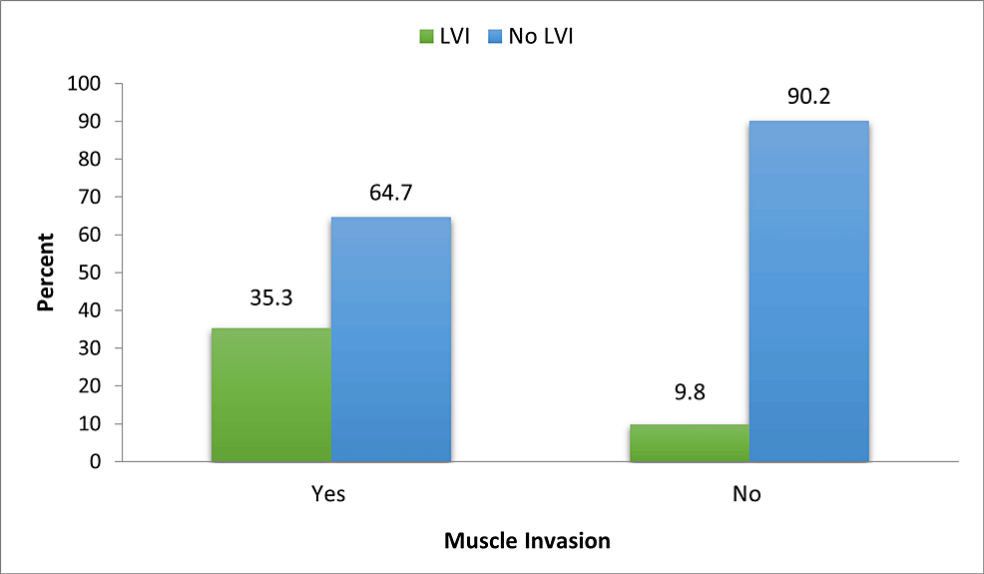
Association between muscle invasion of the tumor and LVI LVI, lymphovascular invasion

In this study, patients with high-grade disease on HPE were more frequently associated with LVI than those with low-grade disease (30% vs 4%). This association was found to be statistically significant with a p-value of 0.010, as seen in Table 5 and Figure 4. A total of 41 (54.7%) patients had non-muscle-invasive bladder tumors and 34 patients (45.3%) were found to have muscle-invasive tumors. Twelve patients with muscle-invasive bladder tumors had LVI identified on their HPE, while only four with non-muscle-invasive tumors had LVI. This association is statistically significant with a p-value of 0.007 (Table 5 and Figure 5).

**Table 5:** Association between grade and muscle invasion of the tumor with LVI LVI, lymphovascular invasion

		With LVI (n=16)	No LVI (n=59)	Total (n=75)	P-value
Tumor grading	High	15 (30%)	35 (70%)	50 (66.7%)	0.010
	Low	1 (40%)	24 (96%)	25 (33.3%)	
Muscle invasion	Present	12 (35.3%)	22 (64.7%)	34 (45.3%)	0.007
	Absent	4 (9.8%)	37 (90.2%)	41 (54.7%)	

## Discussion

Bladder carcinoma is the most common neoplasm of the urinary system. Transurethral resection of bladder tumor (TURBT) is considered the standard procedure for diagnosis, staging, and risk classification of bladder tumors. Further management of bladder tumors depends on the histology, grade, and stage of the disease. LVI is a crucial step in the initiation of tumor dissemination and metastasis. It is well documented in various studies that LVI is a significant poor prognostic factor in bladder tumors [5,6]. The ability to predict and identify LVI in bladder tumors will help us manage such patients more aggressively.

The purpose of our clinical study was to attempt to predict LVI by observing and analyzing various preoperative, intraoperative, and histopathological factors and its association with LVI.

The incidence of LVI in our study was 21.33 %. Mari A et al., in their systemic review and meta-analysis of 33 studies included a total of 6194 patients [7]. LVI was observed in 1069 patients (17.3 %). In a retrospective study done by Yoneda K et al., 50 out of 217 patients had LVI with an incidence of 23% [8]. Another systemic analysis done by Kim et al. in 2014 found that 18.6% of the total study population had LVI [9].

In our study, there was no statistically significant difference between the mean ages of either group. The mean age was 68.19 years in the group with LVI and 64.14 years in the group without LVI with SDs of 11.61 and 11.81, respectively. Our observations were comparable to findings noted by Yoneda K et al, with respect to age distribution [8]. In a prospective study done by Fukumoto et al, the majority of the patients were men (98 (84.5%)) and women were 18 (15.5%) [10]. Sha et al also had 83.2% men and 16.8% women in their study of the impact of LVI on recurrence and progression rates in patients with pT1 urothelial carcinoma of the bladder after transurethral resection [11]. Similarly, 60 (80%) patients were men, and 15 (20%) patients were women in our study.

Our study population was predominantly smokers 49 (65.3%). Twenty-six patients (34.7%) were non-smokers. Twelve out of 49 smokers (24.5%) and four out of 26 non-smokers (15.4%) were noted to have LVI on their HPE. This association was not statistically significant (p-value=0.360).

It is well known that 80-90% of patients with bladder tumors present with single or multiple episodes of hematuria. Likewise, 61 (81.3%) of our subjects presented with hematuria while only 14 (18.7%) had no hematuria. Only 14 patients with a history of hematuria had LVI while the rest had no LVI. In our study, there was no significant association between hematuria and LVI on HPE (p-value=0.475). Unlike our study, Yoneda K et al. found a significant association between gross hematuria and LVI (p-value=0.04). 42 out of 50 (84%) patients with gross hematuria were noted to have LVI [8].

Tumor number and size had no significant association with LVI in our study. With respect to tumor size, most of the study subjects (50.7%) had tumor sizes ranging from 2-5 cm. No tumor of size less than 2 cm had LVI on HPE. We observed that the incidence of LVI increased as the tumor size increased, but this association was not statistically significant (p-value=0.104). Similarly, in a retrospective study done by Ukai R et al., there was no significant association between tumor size and LVI (p-value=0.063) [12].** **Tumor appearance had a significant association with LVI. Fifty-four subjects in our study had sessile tumors. Fifteen out of them (27.8%) had positive LVI. While only one out of 21 patients (4.8%) with pedunculated tumors had positive LVI. Remzi M et al. in their multi-institutional study concluded that LVI was positively associated with sessile architecture of upper tract urothelial tumors [13].

It is widely known that the grade of bladder tumors is an important prognostic factor. High-grade tumors have increased chances to recur and progress to muscle invasion when compared to low-grade tumors. Our study found that the grade of the tumor on HPE significantly correlated with LVI. Fifteen of 50 patients (30%) who had high-grade tumors on HPE also had LVI on their HPE. On the contrary only one of 25 patients (4%) with low-grade tumors had LVI. Yoneda K et al. and Sha et al. concluded in their respective studies that LVI is significantly higher in patients with high-grade tumors and high-grade tumors are more commonly found in patients with LVI [8,11]. Similarly, Bolenz C et al. have also concluded that LVI was significantly associated with increasing tumor grade [14].

Muscle invasion (muscularis propria invasion) is the most important determining factor for further management of bladder tumors after TURBT. In our study, 34 (45.3%) and 41 (54.7%) patients had muscle-invasive and non-muscle-invasive tumors at HPE, respectively. While 12 (35.3%) patients of the muscle-invasive tumors had LVI, only four (9.8%) patients with NMIBC showed LVI. This association between muscle invasion and LVI was found to be statistically significant in our study (p-value=0.007). The incidence rate of LVI at radical cystectomy was found to be ranging from 30% to 50% in the literature [15]. In a comparative study done by Kunju LP et al., LVI at TURBT was more often found in pT2 or greater groups compared to the pT1 group (30% vs 8%) [16].

The main limitation of our study is the sample size. The sample size is small. A larger sample size would have given stronger and more significant results. 

## Conclusions

In our prospective study, we observed that LVI of bladder tumors at first TURBT is significantly associated with tumor grade (high grade vs. low grade), tumor appearance (sessile vs pedunculated), and depth of invasion of the tumor (muscle-invasive vs. non-muscle-invasive). We also noted that LVI was observed in more men than women. This can be attributed to the increased incidence of bladder tumors in men. Though a few variables, viz., smoking and hematuria did not have a statistically significant association with LVI, it was found more commonly occurring in patients who are smokers and who presented with hematuria. Moreover, as the tumor size increased, LVI prevalence increased in our study. We conclude that these factors can be used as reliable predictors of LVI of bladder tumors at their first TURBT.

## References

[1] Mishra V, Balasubramaniam G (2021). Urinary bladder and its associated factors - an epidemiological overview. Indian J Med Sci.

[2] Edge SB, Compton CC (2010). The American Joint Committee on Cancer: the 7th edition of the AJCC cancer staging manual and the future of TNM. Ann Surg Oncol.

[3] Chang SS, Boorjian SA, Chou R (2016). Diagnosis and treatment of non-muscle invasive bladder cancer: AUA/suo guideline. J Urol.

[4] Lotan Y, Gupta A, Shariat SF (2005). Lymphovascular invasion is independently associated with overall survival, cause-specific survival, and local and distant recurrence in patients with negative lymph nodes at radical cystectomy. J Clin Oncol.

[5] Streeper NM, Simons CM, Konety BR, Muirhead DM, Williams RD, O'Donnell MA, Joudi FN (2009). The significance of lymphovascular invasion in transurethral resection of bladder tumour and cystectomy specimens on the survival of patients with urothelial bladder cancer. BJU Int.

[6] Branchereau J, Larue S, Vayleux B, Karam G, Bouchot O, Rigaud J (2013). Prognostic value of the lymphovascular invasion in high-grade stage pT1 bladder cancer. Clin Genitourin Cancer.

[7] Mari A, Kimura S, Foerster B (2019). A systematic review and meta-analysis of the impact of lymphovascular invasion in bladder cancer transurethral resection specimens. BJU Int.

[8] Yoneda K, Utsumi T, Wakai K (2020). Preoperative clinical predictors of lymphovascular invasion of bladder tumors at transurethral resection pathology. Curr Urol.

[9] Kim HS, Kim M, Jeong CW, Kwak C, Kim HH, Ku JH (2014). Presence of lymphovascular invasion in urothelial bladder cancer specimens after transurethral resections correlates with risk of upstaging and survival: a systematic review and meta-analysis. Urol Oncol.

[10] Fukumoto K, Kikuchi E, Mikami S, Miyajima A, Oya M (2016). Lymphovascular invasion status at transurethral resection of bladder tumors may predict subsequent poor response of T1 tumors to bacillus Calmette-Guérin. BMC Urol.

[11] Sha N, Xie L, Chen T (2015). Impact of lymphovascular invasion on recurrence and progression rates in patients with pT1 urothelial carcinoma of bladder after transurethral resection. Onco Targets Ther.

[12] Ukai R, Hashimoto K, Nakayama H, Iwamoto T (2017). Lymphovascular invasion predicts poor prognosis in high-grade pT1 bladder cancer patients who underwent transurethral resection in one piece. Jpn J Clin Oncol.

[13] Remzi M, Haitel A, Margulis V (2009). Tumour architecture is an independent predictor of outcomes after nephroureterectomy: a multi-institutional analysis of 1363 patients. BJU Int.

[14] Bolenz C, Herrmann E, Bastian PJ (2010). Lymphovascular invasion is an independent predictor of oncological outcomes in patients with lymph node-negative urothelial bladder cancer treated by radical cystectomy: a multicentre validation trial. BJU Int.

[15] Abufaraj M, Shariat SF, Foerster B (2018). Accuracy and prognostic value of variant histology and lymphovascular invasion at transurethral resection of bladder. World J Urol.

[16] Kunju LP, You L, Zhang Y, Daignault S, Montie JE, Lee CT (2008). Lymphovascular invasion of urothelial cancer in matched transurethral bladder tumor resection and radical cystectomy specimens. J Urol.

